# Challenges in the integration of palliative care for patients with hematologic malignancies: an analysis of the surprise question in a prospective cohort study

**DOI:** 10.1590/1516-3180.2024.0263.29012025

**Published:** 2025-05-02

**Authors:** Alini Maria Orathes Ponte Silva, Diego Lopes Paim Miranda, David Pereira Ferreira, Camilla Correia de Araujo Pereira Campos, Edvan de Queiroz Crusoé, Felipe Feistauer Gomes, Thiago Favano, Marco Aurélio Salvino

**Affiliations:** I Postgraduate Program in Medicine and Heath, Professor Edgard Santos University Hospital, Medical School, Universidade Federal da Bahia (UFBA), Salvador (BA), Brazil.; II Postgraduate Program in Medicine and Heath, Professor Edgard Santos University Hospital, Medical School, Universidade Federal da Bahia (UFBA), Salvador (BA), Brazil.; III Medical School, Universidade Federal da Bahia (UFBA), Salvador (BA), Brazil.; IV Postgraduate Program in Medicine and Heath, Professor Edgard Santos University Hospital, Medical School, Universidade Federal da Bahia (UFBA), Salvador (BA), Brazil.; V Edgard Santos University Hospital, Universidade Federal da Bahia (HUPES-UFBA), Salvador (BA), Brazil.; VI Postgraduate Program in Medicine and Heath, Faculty of Pharmacy, Universidade Federal da Bahia (UFBA), Salvador (BA), Brazil.; VII School of Pharmaceutical Sciences (FCF), Universidade de São Paulo (USP), São Paulo (SP), Brazil. Mink Therapeutics, USA.; VIII Postgraduate Program in Medicine and Heath, Medical School, Universidade Federal da Bahia (UFBA), Salvador (BA), Brazil.

**Keywords:** Palliative care, Hematologic neoplasms, Advance care planning, Prognoses, Surprise question, Mortality, Oncohematology, Hematologic neoplasms

## Abstract

**BACKGROUND:**

The Surprise Question (SQ), “Would I be surprised if this patient were to die in the next 12 months?”, identifies patients at high risk of death who might benefit from palliative care (PC). However, little is known about its application in oncohematology.

**OBJECTIVES:**

To evaluate the performance of the SQ among inpatients with hematologic malignancies.

**DESIGN AND SETTING:**

A prospective cohort study was conducted between September and December 2021, including patients admitted to the Hematology Ward of the University Hospital in Salvador, Brazil.

**METHODS:**

Physicians answered the SQ (not surprised (SQ+) or surprised (SQ–)). Mortality data were assessed after one year.

**RESULTS:**

Eighty-one patients were included (56% SQ+ and 44% SQ–). At study closure, 36 patients (44%) had died. Median survival was 10.8 months (95%CI = 9.7–11.8) for SQ– and 5.6 months (95%CI = 4.1–7.1) for SQ+. Sensitivity was 86.1%, specificity 68.9%, positive predictive value 68.8%, negative predictive value 86.1%, and accuracy 76.5%. At the time of the interview, only 15 (18.5%) patients had consulted a PC specialist. By the study’s end, 48% had been referred to PC. These patients had poorer performance status (82% vs. 40%, P < 0.001) and more advance care planning records (87% vs. 14%, P < 0.001).

**CONCLUSIONS:**

Despite the prognostic uncertainty of hematologic malignancies, the SQ effectively estimates mortality and serves as a valuable tool for early PC integration in oncohematology.

## INTRODUCTION

Advances in the treatment of hematologic malignancies through targeted therapies and novel hematopoietic procedures have increased overall survival.^1^ However, a substantial proportion of these patients develop refractory disease and experience a high burden of physical and psychological symptoms, leading to impaired quality of life.^2,3^ There is growing recognition that palliative care (PC) should be integrated alongside curative treatments in cases of poor prognosis, following a comprehensive, patient-centered approach that considers existential, psychosocial, and clinical needs.^2-4^ The integration of PC has demonstrated numerous benefits, including improved advance care planning (ACP) , enhanced quality of life, reduced distressing symptoms, and greater satisfaction with care.^5^ However, existing evidence highlights significant barriers to implementing PC in hematologic settings.^4-6^ A key challenge is determining the optimal timing for initiating PC, with a notable obstacle being the high level of prognostic uncertainty. The trajectory of hematologic malignancies is inherently unpredictable, often characterized by rapid deterioration, increased risk of serious complications, and unfavorable outcomes.^3^

However, hematologic malignancies are also marked by the potential for cure, even in advanced stages of disease. This “rollercoaster” trajectory presents unique physical and psychological challenges, and the lack of patient-centered prognostic tools contributes to an existing gap in the provision of PC for these patients.^3^ While early integration of PC is recommended based on patient needs, the availability of prognostic information enables healthcare professionals to determine the appropriate time to initiate open and honest discussions with patients and their families about goals of care, aligning technical knowledge, expectations, values, and preferences in shared decision-making.^7^ The medical team, patients, and their families require support in understanding the therapeutic plan and developing ACP,^8^ even in the face of uncertainty. The cost of uncertainty often results in a significant delay in seeking PC. Practical triggers are essential to facilitate these discussions and promote appropriate referral to PC specialists.^4,9^ The Surprise Question (SQ), “Would I be surprised if this patient were to die in the next 12 months?”, is a screening tool used to identify patients who may benefit from PC.^10^ It is a simple and feasible instrument for intuitively estimating mortality in patients with advanced disease.^11,12^ A “not surprised” response should prompt further screening for PC needs.^6,10,13^

In 2016, the American Society of Clinical Oncology (ASCO) endorsed the SQ in its guidelines on integrating PC into standard cancer treatment^14^ and multiple studies have evaluated its application in solid tumors.^11,13,15^ However, the nonlinear disease trajectory typical of hematologic malignancies raises concerns regarding whether the SQ is an appropriate tool for identifying PC needs in oncohematology.^16^ Despite this, little is known about its application in the context of hematologic malignancies.

## OBJECTIVE

This study aimed to evaluate the performance of SQ in predicting one-year mortality in patients hospitalized with hematologic malignancies and to describe the utilization of PC services in this population.

## METHODS

### Study design

This was a prospective cohort study conducted in the hematology ward of the Professor Edgard Santos University Hospital (HUPES) in Salvador, Brazil. This institution is a well-established academic medical center and a public university hospital specializing in oncological treatments and bone marrow transplantations. HUPES provides PC services through a consultative model, in which PC specialists are called upon to provide expert opinions and, when necessary, collaborate with the clinical teams to support decision-making. During clinical sessions in the hematology ward, physicians assisting hospitalized patients were individually invited to participate in the study. We prospectively included adult patients (age ≥ 18 years) diagnosed with hematologic malignancies who were admitted to the hematology ward of HUPES from September to December 2021 using a convenience sampling method. Only first-time hospital admissions were included; subsequent hospitalizations of the same patients were excluded. All participants, including both patients and physicians, provided written informed consent.

### Data collection

For each patient, the lead physician was asked the SQ (n = 8) and provided one of two responses: “Yes, I would be surprised” or “No, I would not be surprised.” Patients for whom physicians would not be surprised if they died within the next year were coded as SQ+, whereas those for whom physicians would be surprised were coded as SQ–. Patient characteristics and clinical data were extracted from electronic medical records by a single researcher. The analyzed variables included sociodemographic data (age and sex), primary hematologic diagnosis, general disease stage (induction/consolidation, remission, refractoriness, or relapse), use of disease-modifying therapy (surgery, chemotherapy, radiotherapy, immunotherapy, targeted therapy, hormone therapy, bone marrow transplantation, and other treatments), referral to the PC service, and functionality according to the Eastern Cooperative Oncology Group (ECOG) scale.^17^ Twelve months after administering the SQ, a medical record review was conducted to verify mortality status, ACP records, referral requests, and PC consultation reports. The date of the first PC evaluation and the reasons for referral, as pre-established by the PC team, were documented. These reasons included symptom control, decision-making support, development of ACP, and/or early referral.

If a patient had died, additional data were collected, including date and place of death and the use of artificial supportive therapies (orotracheal intubation, dialysis, or vasoactive drugs) in the last 30 days of life. Mortality for all selected patients was confirmed through a review of medical records, a search on the Bahia Civil Registry website https://tjba.jus.br/registrocivil/consultaPublica/search (death registration website in Bahia, Brazil), or telephone call.

### Statistical analysis

The database and descriptive analysis were created using SPSS software (SPSS Inc., Chicago, IL, United States), version 18.0 for Windows. The results are presented in tables. Categorical variables are expressed as frequencies and percentages. Continuous variables with a normal distribution are expressed as means and standard deviations (SD), while those with a non-normal distribution are reported as medians and interquartile ranges. The normality of numerical variables was assessed using descriptive statistics, graphical analysis, and the Shapiro-Wilk test. For comparisons between dichotomous variables (death and SQ response) numerical variables, the independent t-test was applied for normally distributed data, while the Mann-Whitney U test was used for data with a skewed distribution. For comparisons between these variables and categorical variables, the chi-square test was used. When the expected frequency in any category was fewer than five individuals, Fisher’s exact test was applied. To assess the accuracy of the Surprise Question in predicting mortality, the following values were calculated along with their 95% confidence intervals (CIs): sensitivity, specificity, positive predictive value, negative predictive value, positive likelihood ratio, negative likelihood ratio, prevalence, and overall accuracy. Survival analysis was performed using the Kaplan-Meier method, and comparisons of survival curves between SQ responses were conducted using the log-rank test.

### Ethical considerations

This study was approved by the Ethics Committee of HUPES/Medical School of the Federal University of Bahia on August 24, 2021 (CAAE: 50366121.1.0000.0049). The research was conducted in accordance with the Declaration of Helsinki and Good Clinical Practice guidelines.

## RESULTS

Between September and December 2021, 139 patients were admitted. Some patients had multiple hospitalizations. However, only their first hospitalization was considered. Eleven patients were excluded: five had benign diseases, five had solid neoplasms, and one was under 18 years of age. Of the 89 patients who met the inclusion criteria and were potentially eligible, 84 provided informed consent to participate in the study. Among these 84 participants, three were excluded due to missing data.

The demographic and clinical characteristics of the 81 participants, stratified by their SQ response, are presented in Table 1. The mean age of participants was 47.8 years, and the majority were female (58%). Patients had various diagnoses of hematologic malignancies: 29 (35.8%) had acute leukemia, 19 (23.5%) had lymphoma, 27 (33.3%) had multiple myeloma, and six (7.4%) had myelodysplastic syndrome. A total of 50 patients (61.7%) had advanced disease (stage III/IV lymphoma, or relapsed/refractory acute leukemia or multiple myeloma). Additionally, 49 patients (60.5%) had poor performance status (ECOG 3 or 4), and most patients (60.5%) were receiving disease-modifying therapy.

**Table 1:** Characteristics of patients with hematologic malignancies admitted to the hematology Ward of a University Hospital, stratified by physicians? responses to the Surprise Question (SQ). Salvador, Brazil, September to December 2021.

Variables	Total (n = 81)	SQ+ (n = 45)	SQ- (n = 36)	P value
Age (Years; mean ± SD)	47.8 ±16.6	50.3 ±18.1	44.6 ±14.1	0.128*
**Sex; n (%)**				
Female	47 (58)	26 (57.8)	21 (58.3)	0.960**
Male	34 (42)	19 (42.2)	15 (41.7)	
**Hematologic Diagnosis; n (%)**				
Acute Leukemia	29 (35.8)	22 (48.9)	7 (19.4)	0.006**
Lymphoma	19 (23.5)	08 (17.8)	11 (30.6)	0.177**
Multiple Myeloma	27 (33.3)	13 (28.9)	14 (39)	0.343**
Myelodysplastic Syndrome	6 (7.4)	02 (4.4)	04 (11)	0.399***
**Functionality by ECOG scale; n (%)**				
ECOG 0 –2	32 (39.5)	10 (22.2)	22 (61.2)	< 0.001**
ECOG 3 –4	49 (60.5)	35 (77.8)	14 (38.8)	
Use of Disease-Modifying Therapy; n (%)	49 (60.5)	27(60.0)	22 (61.1)	0.919**
Referral to the PC service by interview date, n (%)	15 (18.5)	15 (100)	0 (0.0)	< 0.001***
Referral to the PC service during any hospitalization; n (%)	39 (48.1)	35 (77.8)	4 (11.1)	< 0,001***
Death within 12 months; n (%)	36 (44.4)	31 (68.9)	5 (13.9)	< 0.001***

SQ+ = not surprised, indicates that the physician would not be surprised if the patient died within the next year; SQ- = surprised, indicates that the physician would be surprised if the patient died within the next year; SD = standard deviation; n = number of patients; ECOG = Eastern Cooperative Oncology Group; PC = palliative care;

*T-tests

**chi-square tests

*** Fisher?s exact test.

Physicians responded “No, I would not be surprised” (SQ+) for 45 patients (55.66%) and “Yes, I would be surprised” (SQ−) for 36 patients (44.44%). There were no significant differences in age or sex between the two groups. Compared to patients in the SQ− group, those in the SQ+ group were more likely to have a diagnosis of acute leukemia (48.9% vs. 19.4%, P < 0.006), had significantly worse functionality (ECOG 3–4 in 77.8% vs. 38.9%, P < 0.001), and were more frequently referred to the PC team either by the time of the interview (100% vs. 0%, P < 0.001) or during any subsequent hospitalization (77.8% vs. 11.1%, P < 0.001). After one year, 36 patients had died, resulting in an overall mortality rate of 44.4%. Mortality among those in the SQ+ group was 68.9%, while mortality in the SQ− group was 13.9% (P < 0.001). Participants in the SQ+ group were 13.73 times more likely to die within one year (95%CI = 4.4–42.8) compared to those in the SQ− group.

Table 2 presents the Surprise Question analysis in predicting 12-month mortality in hospitalized patients with hematologic malignancies. The sensitivity of the test was 86.1% (95%CI = 70.5–95.33%), and the specificity was 68.9% (95%CI = 53.35–81.83%). The positive predictive value was 68.8% (95%CI = 58.44–77.71%), the negative predictive value was 86.11% (95%CI = 72.87–93.47%), and the overall accuracy was 76.5% (95%CI = 65.82–82.25%).

**Table 2:** Performance of the Surprise Question in predicting 1-year mortality in hospitalized patients with hematologic malignancies. Salvador, Brazil

	Value (%)	95%CI
Sensitivity	86.11%	70.5 – 95.33
Specificity	68.89%	53.35 – 81.83
Positive Likelihood Ratio	2.77	1.76 – 4.36
Negative Likelihood Ratio	0.20	0.09 – 0.47
Prevalence of Mortality	44.44%	33.4 – 55.91
Positive Predictive Value	68.89%	58.44 – 77.71
Negative Predictive Value	86.11%	72.87 – 93.47
Overall Accuracy	76.54%	65.82 – 85.25

CI = confidence interval.

**Figure 1:**
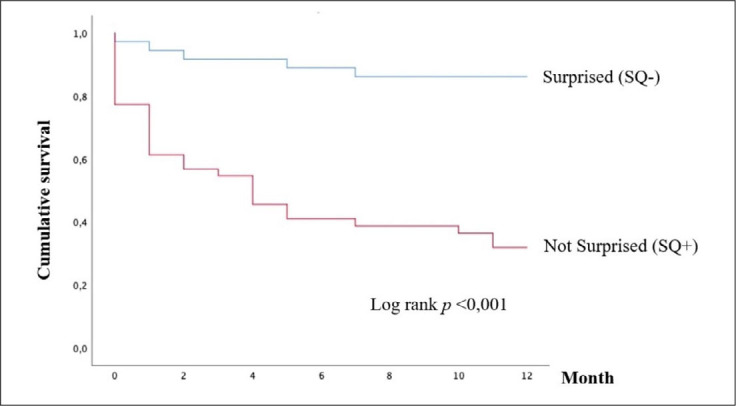
Kaplan-Meier survival curve of patients with hematologic malignancies, Stratified by physicians’ response to the Surprise Question (SQ). Salvador, Brazil, September to December 2021.

Figure 1 shows the comparison of 12-month survival between SQ+ and SQ− responses using the Kaplan-Meier survival curve. A higher survival rate was observed in the SQ− group, with a statistically significant difference. The estimated mean survival time was 10.8 months (95%CI = 9.7–11.8) for the SQ− group and 5.6 months (95%CI = 4.1–7.1) for the SQ+ group.

### Secondary results

At the time of the interview, only 15 patients (18.5%) had been referred to PC services. By the end of 12 months, 39 patients (48.1%) had been referred. Table 3 presents the characteristics of patients based on whether they were referred to PC within one year following the SQ interview. The main reasons for PC referral were symptom control and the development of ACP (76.92%). Only five patients (12.8%) were referred at earlier stages of the disease, and four patients (10.2%) were referred exclusively for decision-making assistance.

**Table 3:** Characteristics of patients with hematologic malignancies admitted to the hematology Ward of a University Hospital, Stratified by follow-up/referral to the palliative care (PC) service. Salvador, Brazil, September to December 2021

Variables	Not Referred to PC Service (n = 42)	Referred to PC Service (n = 39)	P value
Age (years; mean ± SD)	46.8 ± 15.6	48.7 ± 17.7	0.607*
**Sex; n (%)**			
Female	26 (61.9)	21 (53.8)	0.463**
Male	16 (38.1)	18 (46.2)	
**Hematologic diagnosis; n (%)**			
Acute Leukemia	10 (23.8)	19 (48.7)	0.019**
Lymphoma	12 (28.6)	7 (17.9)	0.260**
Multiple Myeloma	17 (40.5)	10 (25.6)	0.157**
Myelodysplastic Syndrome	3 (7.1)	3 (7.7)	0.925**
**Functionality (ECOG scale); n (%)**			
ECOG 0 –2	25 (59.5)	7 (17.9)	< 0.001***
ECOG 3 –4	17 (40.5)	32 (82.1)	
**Surprise Question (SQ); n (%)**			
SQ +	10 (23.8)	35 (89.7)	< 0.001***
SQ–	32 (76.2)	4 (10.3)	
**Use of Disease-Modifying Therapy; n (%)**	26 (61.9)	23 (59.0)	0.787**
**Disease Stage; n (%)**			
Induction/Consolidation	18 (42.9)	13 (33.3)	0.378**
Progression/Relapse/Refractory	24 (57.1)	26 (66.7)	
**End-of-life variables; n (%)**			
Advance Care Planning Registry	6 (14.3)	34 (87.2)	< 0.001***
Death	7 (16.7)	29 (74.3)	< 0.001***
**Death location; n (%)**			
Ward	1 (14.3)	11 (37.9)	0.585**
Intensive Care Unit	5 (71.4)	13 (44.8)
Home	0 (0.0)	2 (6.9)
Ambulance	0 (0.0)	1 (3.4)
Basic Healthcare Unit	1 (14.3)	02 (6.9)
Use of Artificial Life-Support Therapy in the last 30 days of life; n (%)	4 (57.1)	12 (41.1)	0.451**
Median follow-up time between SQ assessment and death (days)	365 (7-365)	130 (0-365)	< 0.001****

PC = palliative care; SD = standard deviation; n = number; ECOG = Eastern Cooperative Oncology Group; SQ+ = not surprised, indicates that the physician would not be surprised if the patient died within the next year; SQ– = surprised, indicates that the physician would be surprised if the patient died within the next year;

*Independent T-test;

** Chi-square test;

*** Fischer?s Exact Test;

****Mann-Whitney test.

When comparing patients who were referred to PC with those who were not, no significant differences were found in age, sex, overall disease stage, or use of disease-modifying therapy. However, patients diagnosed with leukemia were more likely to be referred than those in other diagnostic groups (48.7% vs. 23.8%, P = 0.019). Referred patients also had worse performance status (ECOG 3–4 in 82% vs. 40.4%, P < 0.001), were more frequently classified as SQ+ (89.7% vs. 23.8%, P < 0.001), and had more documented ACP records (87.2% vs. 14.3%, P < 0.001).

Among the 36 patients who died, 29 (74.4%) had received PC follow-up, compared to 7 patients (16.7%) who had not (P < 0.001). Thirty patients (83.3%) died in the hospital, with 18 (50%) passing away in the intensive care unit. Additionally, 16 patients (55.5%) did not receive artificial supportive therapy in the last 30 days of life. The median time from the SQ interview to death was 130 days for patients referred to PC, compared to 365 days for those who were not referred (P < 0.001).

## DISCUSSION

In our study, the SQ was shown to be a promising tool for accurately identifying hospitalized patients with hematologic malignancies who were at risk of dying and, therefore, in need of PC. Among patients who died within a year, 86% were identified by the SQ, and patients with a “surprised” response were significantly less likely to die. Our SQ results align with those of a recent systematic review and meta-analysis, which reported a sensitivity of 83.8% (95%CI = 75.6–92.0%) for cancer patients.^10^ To our knowledge, the study by Hudson et al.^13^ is the only one with a similar population to ours. Their cohort included hospitalized patients with advanced hematologic and solid malignancies, revealing high rates of unmet PC needs. In that study, physicians correctly estimated one-year mortality in 69 patients (68.3%) with stage III/IV lymphoma, relapsed/refractory leukemia, or acute myeloma. Our survival analyses demonstrated that a “wouldn’t be surprised” response was strongly associated with an increased risk of death. A large proportion of our sample consisted of patients with acute leukemia, an aggressive disease with an uncertain trajectory. More than half of the patients had advanced disease and low functionality on the ECOG scale, which aligns with the expected high mortality rate in this population.

At the time of the interview, few patients had been referred to PC, despite a large proportion ultimately dying in the hospital, where specialized PC services were readily available at HUPES. Although physicians anticipated that many of these patients would die within a year, it remains unclear why PC services were underutilized. The literature consistently reports low or late referral rates for patients with hematologic malignancies.^18-20^ In a large study evaluating PC consultation rates at a tertiary cancer center, only 33% of patients with hematologic malignancies received PC, compared to 47% of those with solid malignancies.^20^ Additionally, a survey of 349 American hematologic oncologists found that nearly one-quarter of respondents reported having their first PC discussion only when death was imminent.^21^ The hesitation observed among our physicians may stem from a persistent misperception of PC as exclusively end-of-life care, a misconception that has been well-documented in the literature.^22^

At the conclusion of our study, we reviewed patient records and observed an increase in the number of patients referred to PC services. One possible explanation is that physicians may have been influenced by their responses to SQ, becoming more aware of their patients’ PC needs. Gerlach et al.^23^ reported that hematologic oncologists considered the SQ a valuable tool for stimulating reflection and promoting patient-centered care, facilitating an understanding of the finiteness of life. Since the SQ is an intuitive measure rather than an exact predictive tool, it is particularly useful given the prognostic uncertainty inherent in hematologic malignancies. Another possible explanation for the increased referrals is that they were triggered by clinical deterioration and functional decline. Most patients referred to PC had low functional status (ECOG 3–4), and the primary reason for referral was the need for symptom management and ACP, supporting our hypothesis. While assessing the impact of SQ responses on PC referrals was not the primary aim of this study, future research could further explore this relationship. Notably, most ACP records were documented by PC specialists. It is important to emphasize that ACP is a gradual process, construction and its elements should be introduced progressively, in accordance with each patient’s circumstances.^7^ Furthermore, initiating ACP at an earlier stage does not appear to cause harm.^10^ Due to the poor functional status of patients in the “ SQ+” group, ACP plays a crucial role in helping them articulate and prioritize their care goals in the context of their health status and express their treatment preferences.

To our knowledge, this is one of the first studies to evaluate the accuracy of SQ in hospitalized patients with hematologic malignancies and represents a pioneering study in Latin America. Clinicians can incorporate the SQ into routine clinical assessments as a valuable trigger to initiate timely discussions about goals of care, improve communication, and facilitate referral to PC services. Currently, no other tool for identifying PC needs in patients with hematologic malignancies appears to be as effective and as simple as the SQ. Future research should focus on validating the efficacy of the SQ across different clinical settings and patient subgroups within hematologic malignancies. Additionally, further studies are needed to determine the optimal frequency for applying the SQ as an identification tool and to assess its cost-effectiveness in PC delivery. This study has several limitations. First, the sample size was small and restricted to a single academic center, which serves as a public hospital and a referral center for hematologic diseases. Second, the accuracy of the SQ may vary across specific disease groups, given the distinct trajectories of aggressive and chronic hematologic malignancies. The small number of patients within each hematologic malignancy subtype limited statistical comparisons between disease types. Despite these differences, basic PC needs are common across all hematologic populations, as demonstrated in multiple studies ^24^.

## CONCLUSION

The early integration of PC for patients with hematologic malignancies remains a significant challenge. However, SQ has demonstrated its ability to reliably estimate mortality and serves as a valuable tool, even in the context of the prognostic uncertainty inherent to these diseases. The SQ provides a practical starting point, enabling timely discussions about goals of care and facilitating referrals to specialized PC services. Its use in routine clinical practice may help bridge the gap in PC access for this patient population.
